# Dynamic functional connections in leukoaraiosis patients without cognitive impairment: A pilot study

**DOI:** 10.3389/fnagi.2022.944485

**Published:** 2022-09-01

**Authors:** Xingru Xu, Yu-Chen Chen, Xindao Yin, Taosheng Zuo, Guangkui Feng, Kaixi Xu

**Affiliations:** ^1^Department of Radiology, Affiliated Lianyungang Traditional Chinese Medicine Hospital of Kangda College of Nanjing Medical University, Lianyungang, China; ^2^Department of Radiology, Nanjing First Hospital, Nanjing Medical University, Nanjing, China; ^3^Department of Neurology, Affiliated Lianyungang Traditional Chinese Medicine Hospital of Kangda College of Nanjing Medical University, Lianyungang, China

**Keywords:** leukoaraiosis, cognitive impairments, dynamic connectivity, functional connections, functional MRI

## Abstract

**Purpose:**

Leukoaraiosis (LA) is a major public issue that affects elderly adults. However, the underlying neuropathological mechanism of LA without cognitive impairment requires examination. The present study aimed to explore the dynamic functional network connectivity (dFNC) in LA patients without cognitive impairment.

**Methods:**

Twenty-three patients with LA and 20 well-matched healthy controls were recruited for the present study. Each subject underwent magnetic resonance imaging (MRI) scanning and cognition evaluations. Spatial independent component analysis was conducted to evaluate dynamic functional connectivity. The differences in dFNC were determined and correlated with cognitive performance.

**Results:**

Compared with controls, LA without cognitive impairment showed aberrant dFNC in State 1, involving increased connectivity in the default mode network (DMN) with the executive control network (ECN). In addition, decreased connectivity in the DMN with the salience network (SN) was found in State 3. Furthermore, the decreased number of transitions between states was positively associated with the visuospatial/executive score (Spearman's rho = 0.452, *p* = 0.031), and the longer mean dwell time in State 1 was negatively associated with the Montreal Cognitive Assessment (MoCA) score (Spearman's rho = – 0.420, *p* = 0.046).

**Conclusion:**

These findings enrich our understanding of the neural mechanisms underlying LA and may serve as a potential imaging biomarker for investigating and recognizing the LA at an early stage.

## Introduction

Leukoaraiosis (LA) is a common neuroimaging phenomenon that often occurs in older people (Smith, 2010). There is growing evidence to suggest that the prevalence of magnetic resonance imaging (MRI)-detected LA in people over age 60 years is greater than 30% and increases with age. LA has been recognized to be linked with cognitive dysfunction or dementia and several neurodegenerative disorders. The effects of LA on cognitive function are insidious and may be difficult to detect at the early stage but are crucial. It has been confirmed to be related to cognitive impairment, such as a reduction in the cognitive processing speed, executive function, and visual space function (Van Straaten et al., 2006). Besides that, several studies have found normal cognitive function at the early stage in LA patients (Koga et al., 2009; Wardlaw et al., 2015). However, the potential neuro-mechanism for LA without cognitive dysfunction has been unclear to date. Therefore, the neural substrates underlying LA without cognitive impairment need to be elucidated currently.

Prior neuroimaging studies have demonstrated reduced functional connectivity networks, such as the default mode network (DMN), dorsal attention network (DAN), or other cognitive networks, in LA patients (Li et al., 2015; Chen et al., 2019, 2021). Specifically, decreased causal functional connectivity between the DMN and DAN was observed in LA-associated cognitive impairment (Shi et al., 2020). Together, these results suggest that network alterations may reflect clinically relevant phenomena in LA. However, due to the assumption that functional connectivity is constant during resting-state functional MRI (fMRI) scans, most studies have not considered the important aspects from the dynamic perspective. The temporal dynamics of brain network connections can be detected by analyzing fMRI data (Allen et al., 2014). Aberrant dynamic functional connectivity is linked to specific neurological diseases, such as Alzheimer's disease (Gu et al., 2020), Parkinson's disease (Fiorenzato et al., 2019), presbycusis (Xing et al., 2021), and mild traumatic brain injury (Lu et al., 2021). In addition, several studies of subcortical ischemic vascular disease have investigated altered dynamic functional connectivity (Fu et al., 2019). Nevertheless, alterations in dynamic functional connectivity in early stage LA patients are still unclear. Therefore, this study aimed to identify differences in dynamic functional connectivity in LA patients without cognitive impairment as well as healthy controls (HCs). We hypothesized that LA patients might demonstrate aberrant dynamic functional network connectivity (dFNC) and these dFNC aberrances would correlate with abnormal cognitive performance.

## Materials and methods

### Subjects and clinical data

We recruited 23 LA patients from the Department of Neurology in our hospital and 20 age-, sex-, and education-matched HCs from the local community *via* advertisements. The inclusion criteria for the LA patients were as follows: (1) patients aged between 50 and 80 years old and (2) patients showed LA on MRI scans according to the revised version of the scale of Fazekas et al. (1987). All patients had subcortical white matter hyperintensity (WMH) on T2-weighted images and were diagnosed with ischemic brain diseases (Wardlaw et al., 2013). The exclusion criteria were as follows: (1) cortical or subcortical infarct or hemorrhage, Parkinson's disease, Alzheimer's disease, neurologic disorders, and major illnesses; (2) a history of brain injury, drug addition, smoking, or alcoholic addiction; and (3) MRI contraindications, such as cochlear implants, pacemakers, or prosthetic valves.

### Cognitive evaluation

All subjects underwent standardized clinical neuropsychological evaluations, such as the Mini-Mental State Examination (MMSE) and Montreal Cognitive Assessment (MoCA). All subjects provided written consent before their participation in the study protocol, which was approved by the Medical Research Ethics Committee of Nanjing Medical University.

### Scan acquisition

All functional and structural imaging data were acquired using a 3.0 Tesla Philips MRI equipment (Discovery MR750, General Electric, Milwaukee, WI, USA) using a 32-channel head coil, including 3D-T1 and blood-oxygen-level-dependent (BOLD) sequences. Foam padding was used to reduce head motion, and earplugs were used to attenuate the influence of noise by ≈ 32 dB. Every subject was asked to lie quietly, keep their eyes closed, remain awake, and avoid thinking about anything special during the whole scanning. The parameters of 3D-T1 weighted imaging were as follows: repetition time (TR) = 8.2 ms, echo time (TE) = 3.2 ms, slices = 170, thickness = 1 mm, gap = 0 mm, fractional anisotropy (FA) = 12°, field of view (FOV) = 240 × 240 mm, matrix = 256 × 256. BOLD was acquired using a gradient echo-planar imaging sequence as follows: TR = 2,000 ms, TE = 30 ms, slice = 36, thickness = 4 mm, gap = 0 mm, FA = 90°, FOV = 240 × 240 mm, and matrix = 64 × 64.

### Data processing

Functional data were preprocessed using DPABI (http://rfmri.org/dpabi) and SPM 12 (http://www.fil.ion.ucl.ac.uk/spm) on MATLAB (R2021b). The preprocessing steps of functional data were similar to those in a previous study (Xu et al., 2019), including (1) removing the first 10 time points to minimize the effect of signal instability; (2) slice timing; (3) realignment for head motion correction; (4) segmentation; (5) normalization to a standard template; (6) regressing 6 motion parameters, 6 temporal derivatives and 12 corresponding squared items using the Friston-24 parameter; (7) smoothing with 6-mm full width at half-maximum Gaussian kernel; and (8) detrending and filtering (0.01–0.08 Hz). Subjects with a translational or rotational head motion of >2.0 mm or 2.0° in any direction were excluded.

Then, group spatial ICA was analyzed using the Group ICA fMRI toolbox (GIFT) (Calhoun et al., 2001). The ICA analysis consisted of three stages: data reduction, application of the ICA algorithm, and back reconstruction for each individual subject. A modified version of the minimum description length criterion was adopted to determine the number of components from the aggregate dataset (Li et al., 2007). Single participant spatial or temporally independent maps were then back-reconstructed from the aggregate mixing matrix. This procedure was repeated several times in ICASSO (http://research.ics.tkk.fi/ica/icasso/) to ensure estimation stability. Then, subject-specific spatial maps and time courses were obtained using a back reconstruction approach, and the results were converted to *z* scores. Among the 34 independent components (ICs), 20 ICs were acquired and categorized into 7 RSNs for subsequent analyses according to previous reports.

The dFNC analysis was performed using the temporal dFNC toolkit in GIFT software. The dFNC between ICA time processes was calculated using a sliding time-window approach. The sliding window size was set to a width of 20 and TRs = 40 s with a Gaussian (= 3 TRs), with each step advancing 1 TR, resulting in W = 128 windows. Then, the *k*-means clustering algorithm using the squared Euclidean distance method with 500 iterations and 150 replicate dFNC windows was conducted on the windowed FNC (wFNC) matrices (Malhi et al., 2019). The number of optimal mental states was estimated based on the elbow criterion, which is defined as the ratio of within- to between-cluster distances. Using this method, *k* is 5 in the search window *k* is 2–10 (Damaraju et al., 2014). Two-sample *t*-tests were used to compare each dFNC state between LA and HC with a significance threshold of *p* < 0.05 [false discovery rate (FDR)-corrected]. The temporal properties of dFNC states were analyzed by computing the fraction time and mean dwell time in each state and the number of transitions between states. A two-sample *t*-test was used to analyze the significance of the mean residence time and transition times in the LA and HC groups (*p* < 0.05, FDR corrected).

### Statistical analysis

Differences in demographic and clinical variables between groups were compared using independent sample *t*-tests with SPSS software (version 20.0; SPSS, Chicago, IL). Spearman's correlation analysis was used to assess the relationship among the network metrics, temporal attributes and cognitive score, and controlling for age and gender. The significant *p* value was set at <0.05. Moreover, the effect size and the power for each significant network metric (*p* < 0.05) were also analyzed. The effect size of the group mean difference is measured using Cohen's d with pooled standard deviation (Cohen, 1992). The statistical power of the test with a significance level 0.05 is calculated based on the group means, standard deviations, and sample sizes, using an online power calculator (http://www.powerandsamplesize.com/).

## Results

### Demographic and neuropsychological characteristics

The demographic and cognitive characteristics of both LA patients and HCs are presented in Table 1. No significant group differences were found in age, sex, education, MMSE, or MoCA scores between the LA and HC groups. No significant differences in cognition were observed between LA without cognitive impairment and HCs.

**Table 1:** The detailed information about demographic and neuropsychological data in patients and control participants.

**Characteristics**	**LA patients (*n* = 23)**	**HC (*n* = 20)**	***P*-value**	**Effect size**
Age (years)	64.96 ± 7.87	61.80 ± 5.81	0.147	0.22268
Gender (M:F)	14/9	12/8	0.954	–
Education (years)	10.52 ± 1.93	10.50 ± 1.43	0.967	0.00589
MMSE scores	28.87 ± 1.39	28.70 ± 1.22	0.675	0.06486
MoCA scores	26.48 ± 1.88	25.85 ± 1.73	0.263	0.17177

Data are presented as the Mean ± SD. LA, leukoaraiosis; HC, healthy controls; MMSE, Mini-Mental State Examination; MoCA, Montreal Cognitive Assessment; M, male; F, female.

### Dynamic functional network connectivity analysis

**Figure 1:**
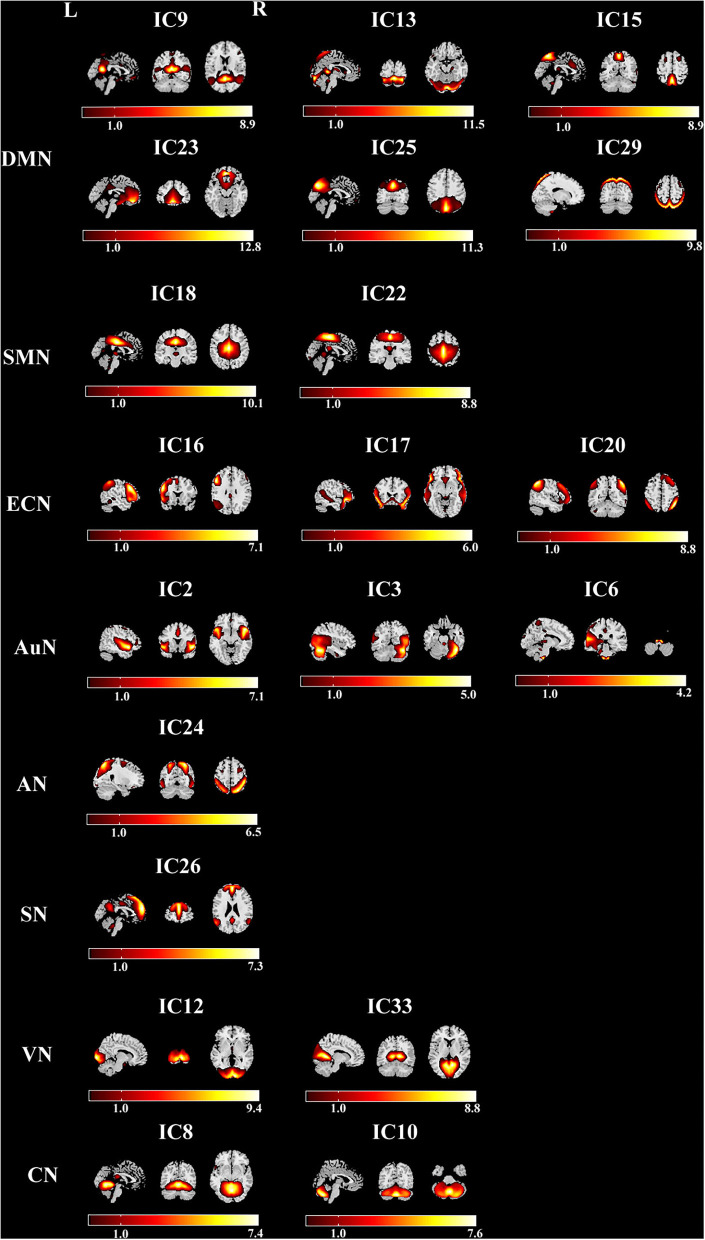
The serial cross sections of the brain representing the eight networks with 20 components in the leukoaraiosis (LA) group. DMN, default mode network; SMN, sensorimotor network; ECN, executive control network; AuN, auditory network; AN, attention network; SN, salience network; VN, visual network; CN, cerebellum network.

Twenty ICs were selected as our networks of interest (Figure 1), which were categorized into the eight resting-state networks: DMN (ICs 9, 13, 15, 23, 25, 29), sensorimotor network (SMN) (ICs 18, 22), executive control network (ECN) (ICs 16, 17, 20), auditory network (AuN) (ICs 2, 3, 6), attention network (AN) (IC 24), salience network (SN) (IC 26), visual network (VN) (ICs 12, 33), and cerebellum network (CN) (ICs 8, 10). The cluster stability/quality was very high (Iq > 0.9) for 20 components.

**Figure 2:**
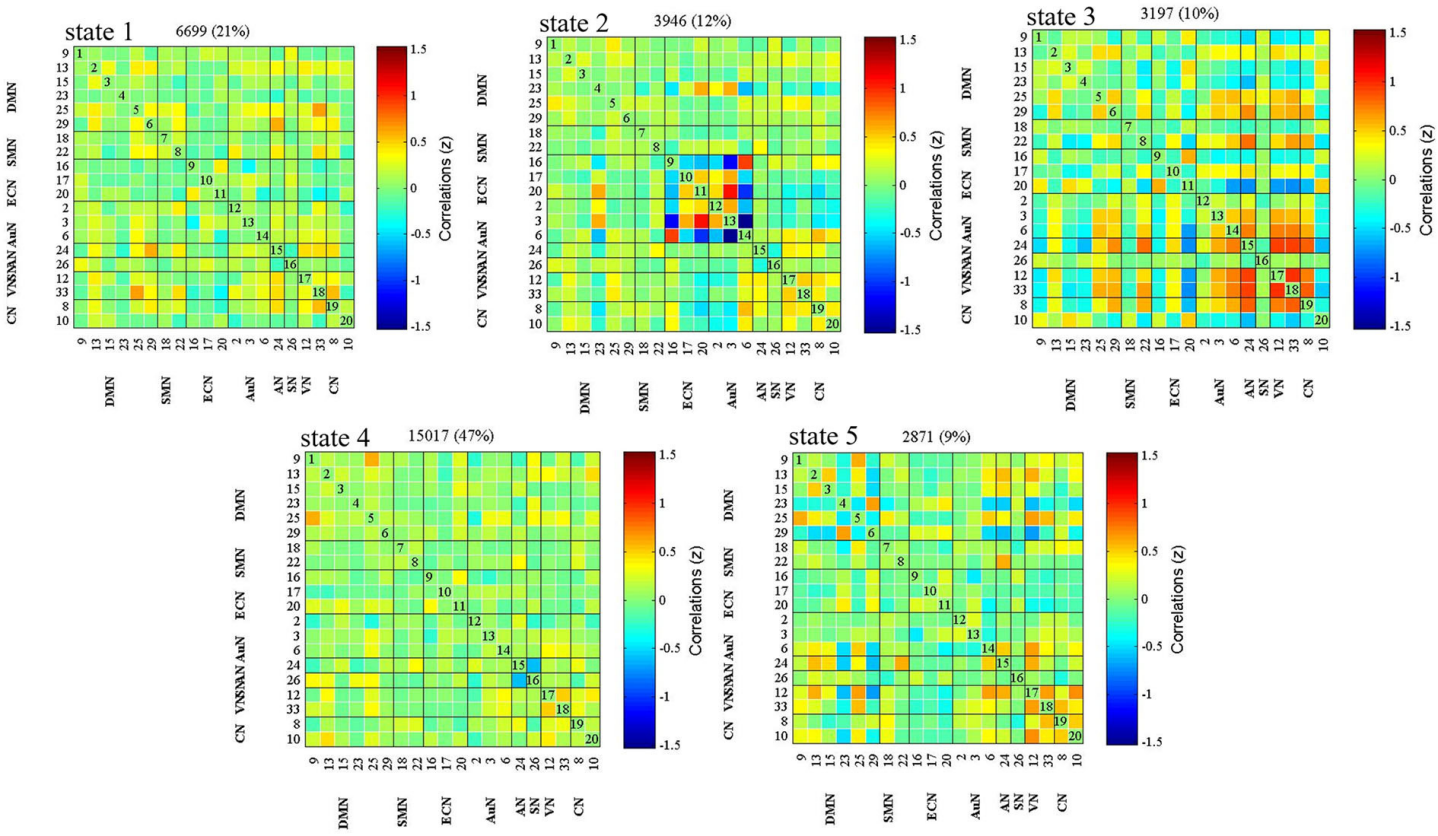
Dynamic functional network connectivity (dFNC) centroids obtained for the five states using the *k*-means algorithm. The total number of occurrences and percentage of total occurrences are listed above in each cluster median.

**Figure 3:**
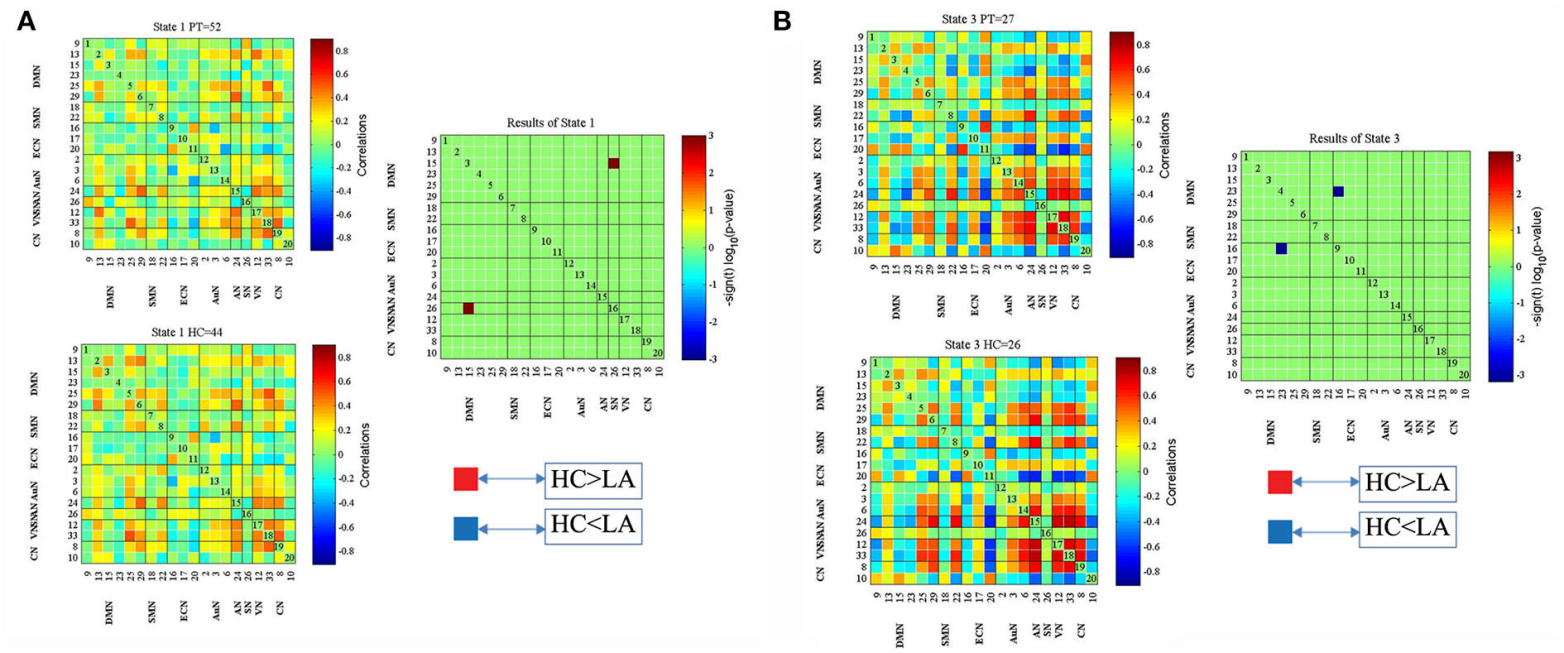
Significant differences in dFNC between the LA and healthy control (HC) groups in State 1 **(A)** and State 3 **(B)**. The top and bottom rows on the left side represent median connectivity matrices in LA and HC, respectively.

Figure 2 displays five common functional connectivity states and corresponding centroids of clusters: the percentages of total occurrences of these five states differed, with State 1 (76.1%), State 2 (23.9%), State 3 (23.9%), State 4 (23.9%), and State 5 (23.9%). The significant differences between the two groups in dFNC were found in State 1 and State 3. Coupling among the two networks differed between groups in State 1, representing the DMN (IC15) and SN (IC26) (HC > LA, *p* < 0.05, FDR correction) (Figure 3A). In State 3, the dFNC between the DMN (IC23) and ECN (IC16) differed between the LA and HC groups (LA > HC, *p* < 0.05, FDR correction) (Figure 3B).

**Figure 4:**
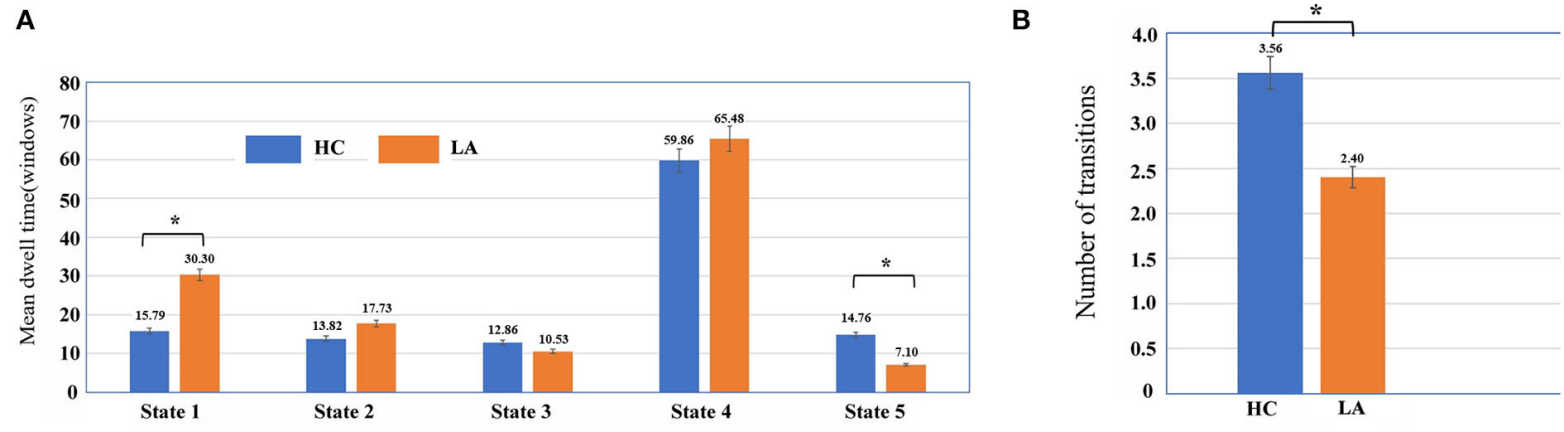
Temporal properties of functional connectivity state analysis in LA and HC groups, including mean dwell time **(A)** and number of transitions **(B)**. Asterisks represent significant group differences (*p* < 0.05, FDR corrected).

The significant group differences were observed in the mean dwell time of the two states (Figure 4). The mean dwell time in State 1 was significantly longer in the LA group than in the HC group (mean ± SD for LA: 38.83 ± 9.96; for HC: 15.79 ± 26.67, *p* < 0.05; power: 0.9935). The mean dwell time in State 5 was significantly shorter in the LA group than in the HC group (mean ± *SD* for LA: 7.10 ± 14.65; for HC: 14.76 ± 27.78, *p* < 0.05; power: 0.3413). Moreover, the number of transitions between states in the LA group was smaller than that in the HC group (mean ± SD for LA: 4.00 ± 2.26; for HC: 5.56 ± 2.25, *p* = 0.017; power: 0.7831). However, no significant group difference in fractional windows in each state was detected (*p* > 0.05).

### Correlation analysis

**Figure 5:**
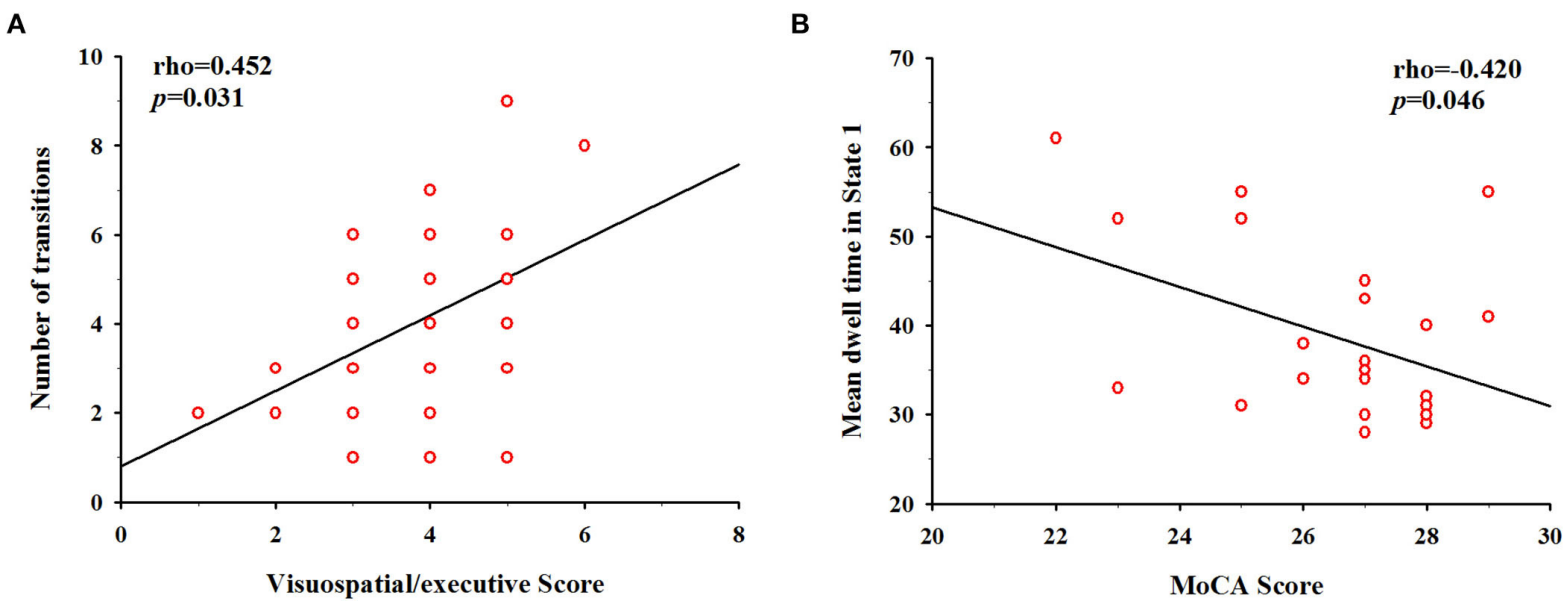
Correlation between cognitive scores and temporal properties in LA patients. **(A)** The number of transitions was positively correlated with the visuospatial/executive score (Spearman's rho = 0.452, *p* = 0.031). **(B)** The mean dwell time in State 1 was negatively correlated with Montreal Cognitive Assessment (MoCA) score (Spearman's rho = −0.420, *p* = 0.046).

In the correlation analysis between dFNC properties and cognitive performance in the LA group (Figure 5), we found that the reduced number of transitions was positively correlated with visuospatial/executive scores (Spearman's rho = 0.452, *p* = 0.031), suggesting a relationship between dynamic state changes and LA patients' visuospatial/executive performance. In addition, the longer mean dwell time in State 1 was negatively correlated with MoCA score (Spearman's rho = – 0.420, *p* = 0.046).

## Discussion

This study evaluated the dFNC abnormalities among LA subgroups and HCs, in order to assess the neural mechanism of LA without cognitive impairment. To our knowledge, most existing studies have focused on LA with cognitive impairment; and our research is the first study to examine the aberrant dFNC and cognitive condition in LA subjects.

Our results suggest increased interactions of the DMN-ECN, along with decreased interactions with the DMN-SN and altered temporal properties. In addition, these aberrations were correlated with cognitive decline in the LA group. Consistent with the results regarding the increased interactions of DMN-ECN, Li et al. showed that LA patients without cognitive impairment exhibited significantly increased amplitude of low-frequency fluctuations (ALFF) in the precuneus and superior frontal gyrus (SFG) compared with normal controls (Li et al., 2015). The DMN, consisting of the precuneus, prefrontal, and temporoparietal junction areas, appears to be one of the key brain networks in the resting state (Raichle et al., 2001). The precuneus is located in the medial region of the parietal lobe and is responsible for memory and executive function (Cavanna and Trimble, 2006). In addition, the SFG plays a pivotal role in attention, execution control, and self-awareness (Li et al., 2013). Therefore, we speculate that LA may disrupt the enhanced interactions within the DMN-ECN leading to network disruption. Furthermore, Chen et al. demonstrated that LA patients showed decreased functional connectivity between the DMN and the SN (Chen et al., 2019), which was similar to our findings. The previous studies also confirmed that the functional connectivity between the DMN and SN might provide a characterization of the cognitive impairment of the LA (Reijmer et al., 2015; Atwi et al., 2018). Thus, we hypothesized that aberrant dFNC between the DMN and other resting-state networks may act as a potential imaging biomarker for early cognitive dysfunction caused by LA, suggesting that aberrant dFNC may exist prior to overt cognitive impairment and symptoms in LA patients.

Different dFNC temporal properties were found in LA patients. The decreased transition between states is applied to distinguish the LA from the HC. Moreover, the dFNC temporal properties are associated with cognitive dysfunction in multiple domains, such as memory, execution, attention, and visuospatial function (Fortenbaugh et al., 2018). Therefore, these observations may indicate functional network vulnerability in LA and emphasize the importance of detecting LA for dFNC temporal properties. Further studies are required to confirm whether aberrant functional segregation and the number of state transitions can act as potential biomarkers for LA.

Several limitations should be acknowledged in our study. First, the current study is observational with a relatively small sample size, which may restrict the statistical power. We did not include the LA patients with cognitive impairment. Further research is needed to expand the sample size and enroll the other types of LA patients. Second, we did not take the duration of LA into consideration; we enrolled LA patients without cognitive impairment to observe early brain functional abnormalities. Thus, future studies detecting dynamic network changes in different courses of LA will be considered. Finally, this study is a cross-sectional experimental design, and a longitudinal study should be conducted to explore the progression of cognitive impairment and the causal relationship between dFNC changes and cognitive performance in LA patients.

## Conclusion

In summary, this preliminary study is the first to focus on the different connections between HCs and LA without cognitive impairment, examining the neural mechanism underlying LA. Our study suggests that temporal functional dynamics may help contribute to early diagnosis. Moreover, this research could provide a potential therapeutic target for LA in the future.

## Data availability statement

The original contributions presented in the study are included in the article/supplementary material, further inquiries can be directed to the corresponding author/s.

## Ethics statement

The studies involving human participants were reviewed and approved by Nanjing Medical University. The patients/participants provided their written informed consent to participate in this study.

## Author contributions

XX and Y-CC collected the fMRI data, performed the analysis and wrote this manuscript. XY helped with data analysis and discussion. TZ helped with recruitment of subjects and data processing. Y-CC designed this experiment and revised this manuscript. GF and KX contributed equally to this work and shared the first authorship. All authors contributed to the article and approved the submitted version.

## Funding

This work was supported by the Jiangsu Provincial Special Program of Medical Science (No. BE2021604) and Natural Science Foundation of Jiangsu Province (No. BK20201118).

## Conflict of interest

The authors declare that the research was conducted in the absence of any commercial or financial relationships that could be construed as a potential conflict of interest. The reviewer JC declared a shared affiliation with the authors to the handling editor at the time of review.

## Publisher's note

All claims expressed in this article are solely those of the authors and do not necessarily represent those of their affiliated organizations, or those of the publisher, the editors and the reviewers. Any product that may be evaluated in this article, or claim that may be made by its manufacturer, is not guaranteed or endorsed by the publisher.

## Abbreviations

LA: Leukoaraiosis
MRI: magnetic resonance imaging
HC: healthy controls
dFNC: dynamic functional network connectivity
DMN: default mode network
ECN: executive control network
SN: salience network
DAN: dorsal attention network
AuN: auditory network
AN: attention network
VN: visual network
CN: cerebellum network
fMRI: functional magnetic resonance imaging
WMH: white matter hyperintensity
MMSE: Mini-Mental State Examination
MoCA: Montreal Cognitive Assessment
TR: repetition time
TE: echo time
FOV: field of view
FA: flip angle
FLAIR: fluid-attenuated inversion recovery
BOLD: blood-oxygen-level-dependent
ALFF: amplitude of low frequency fluctuations
SFG: superior frontal gyrus.
